# *Rol* genes enhance the biosynthesis of antioxidants in *Artemisia carvifolia* Buch

**DOI:** 10.1186/s12870-016-0811-7

**Published:** 2016-06-02

**Authors:** Erum Dilshad, Hammad Ismail, Ihsan-ul- Haq, Rosa Maria Cusido, Javier Palazon, Karla Ramirez-Estrada, Bushra Mirza

**Affiliations:** Department of Biochemistry, Faculty of Biological Sciences, Quaid-i-Azam University, Islamabad, Pakistan; Department of Pharmacy, Quaid-i-Azam University, Islamabad, Pakistan; Laboratorio de Fisiologia Vegetal, Facultad de Farmacia, Universidad de Barcelona, Barcelona, Spain

**Keywords:** *Agrobacterium tumefaciens*, *Artemisia carvifolia* Buch, antioxidant assays, Chalcone synthase, Flavonoids, Phenylalanine ammonia-lyase, *Rol* gene

## Abstract

**Background:**

The secondary metabolites of the *Artemisia* genus are well known for their important therapeutic properties. This genus is one of the valuable sources of flavonoids and other polyphenols, but due to the low contents of these important metabolites, there is a need to either enhance their concentration in the original plant or seek alternative sources for them. The aim of the current study was to detect and enhance the yield of antioxidant compounds of *Artemisia carvifolia* Buch. HPLC analysis was performed to detect the antioxidants. With the aim of increasing flavonoid content, *Rol* gene transgenics of *A. carvifolia* were established. Two genes of the flavonoid biosynthetic pathway, phenylalanine ammonia-lyase and chalcone synthase, were studied by real time qPCR. Antioxidant potential was determined by performing different antioxidant assays.

**Results:**

HPLC analysis of wild-type *A. carvifolia* revealed the presence of flavonoids such as caffeic acid (30 μg/g DW), quercetin (10 μg/g DW), isoquercetin (400 μg/g DW) and rutin (300 μg/g DW). Compared to the untransformed plants, flavonoid levels increased 1.9–6-fold and 1.6–4-fold in *rol B* and *rol C* transgenics, respectively. RT qPCR analysis showed a variable expression of the flavonoid biosynthetic genes, including those encoding phenylalanine ammonia-lyase and chalcone synthase, which were found to be relatively more expressed in transformed than wild-type plants, thus correlating with the metabolite concentration. Methanolic extracts of transgenics showed higher antioxidant capacity, reducing power, and protection against free radical-induced DNA damage. Among the transgenic plants, those harboring *rol B* were slightly more active than the *rol C-*transformants.

**Conclusion:**

As well as demonstrating the effectiveness of *rol* genes in inducing plant secondary metabolism, this study provides insight into the molecular dynamics of the flavonoid accumulation pattern, which correlated with the expression of biosynthetic genes.

## Background

*Artemisia* is a diverse and economically important genus belonging to the family Asteraceae, with over 300 species [1]. This genus is a source of valuable secondary metabolites and essential oils used in the treatment of various diseases [2]. Phenols in general and flavonoids in particular are one of the most important groups of phytochemicals in plants, affecting oxidative stability, appearance, taste and odor. The biological properties shown by these compounds include antioxidant, anti-cancer and anti-aging effects, as well as protection against different heart and immune diseases and brain dysfunction caused by Parkinson’s, Alzheimer’s and Huntington’s diseases [3, 4].

**Fig. 1:**
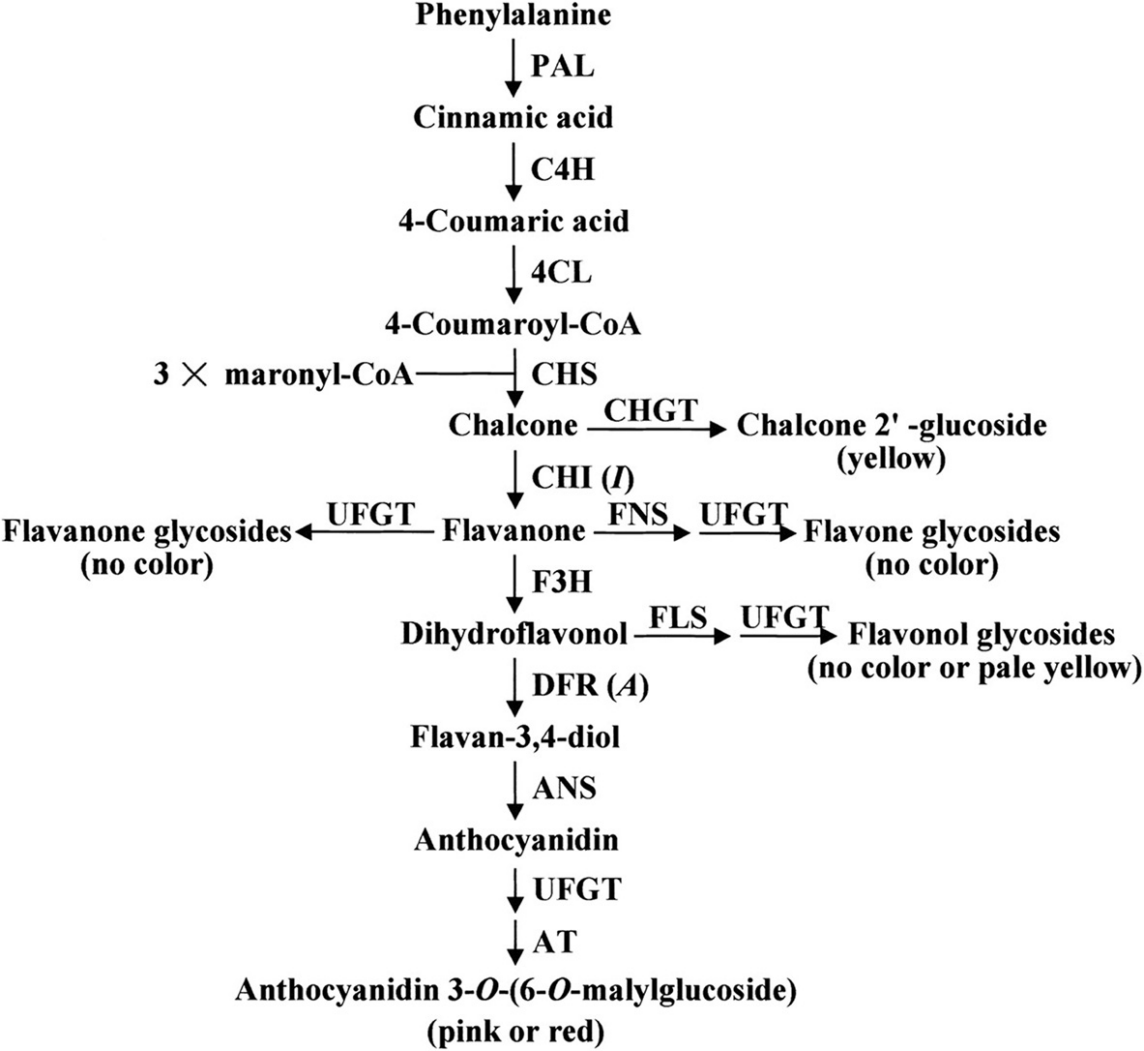
Flavonoids biosynthetic pathway [57]

Flavonoid biosynthesis starts with the amino acid L-phenylalanine [5] and leads to the formation of 4-coumaroyl CoA by the phenylproponoid pathway [6]. The key enzyme of this pathway is phenylalanine ammonia-lyase (*PAL*) [7], others being cinnamate 4-hydroxylase (*C4H*) and 4-coumarate: CoA ligase (*4CL*) [6]. Chalcone synthase (*CHS*), which catalyzes the first committed step of the flavonoid pathway, is involved in the production of naringenin chalcone by combining one coumaroyl CoA molecule with three malonyl CoA molecules. Chalcone isomerase (*CHI*) further isomerizes the chalcone to flavanone and from this step onward the pathway diverges to form diverse classes of flavonoids (Fig. 1).

The *Artemisia* genus is a particularly rich source of flavonoids [8]. While *A. annua* is the most extensively studied species, resulting in the isolation of around 50 flavonoids, these antioxidants have also been detected in other species, including *A. absinthium* L [9], *A. asiatica* [10], *A. Herba-Alb* [11]. However, the flavonoid concentration is usually very low and highly variable, not only among different chemotypes but also in the same plant at different growth stages [12].

Among different strategies used to improve plant secondary metabolite production, recombinant DNA technology has allowed the expression of biosynthetic genes to be altered, and the manipulation of metabolic traits [13]. Several studies show that *rol* genes are powerful activators of secondary metabolism in various plants [14]. Protein of the *rol* A gene binds to DNA and stimulates growth, whereas the *rol B* gene is involved in the regulation of the auxin signal transduction pathway [15] and is a potent inducer of plant secondary metabolism, increasing the resveratrol production in *Vitis amurensis* [16] and anthraquinones in *Rubia cardifolia* [17]. The *rol* C gene encodes cytokinin glucosidase and stimulates the production of many secondary compounds in various plants [18–22].

In previous work, we obtained *rol B* and *rol C* transformants of *A. carvivolia* Buch with a high yield of antimalarial compounds as well as higher transcript levels of biosynthetic genes than the wild-type plant [21]. The objective of the current study was to enhance the content of flavonoids in *Artemisia carvifolia* Buch transgenics after their detection in the wild-type plant. We carried out real time qPCR analysis of flavonoid biosynthetic genes to find a relationship between their expression levels and metabolite concentration. In this regard, two genes of the phenylpropanoid pathway of flavonoid biosynthesis were studied: those encoding *PAL* and *CHS*. The flavonoids were quantified by HPLC, and antioxidant activity was measured by performing different antioxidant assays.

## Results and discussion

### HPLC-DAD-based quantification of flavonoids

**Fig. 2:**
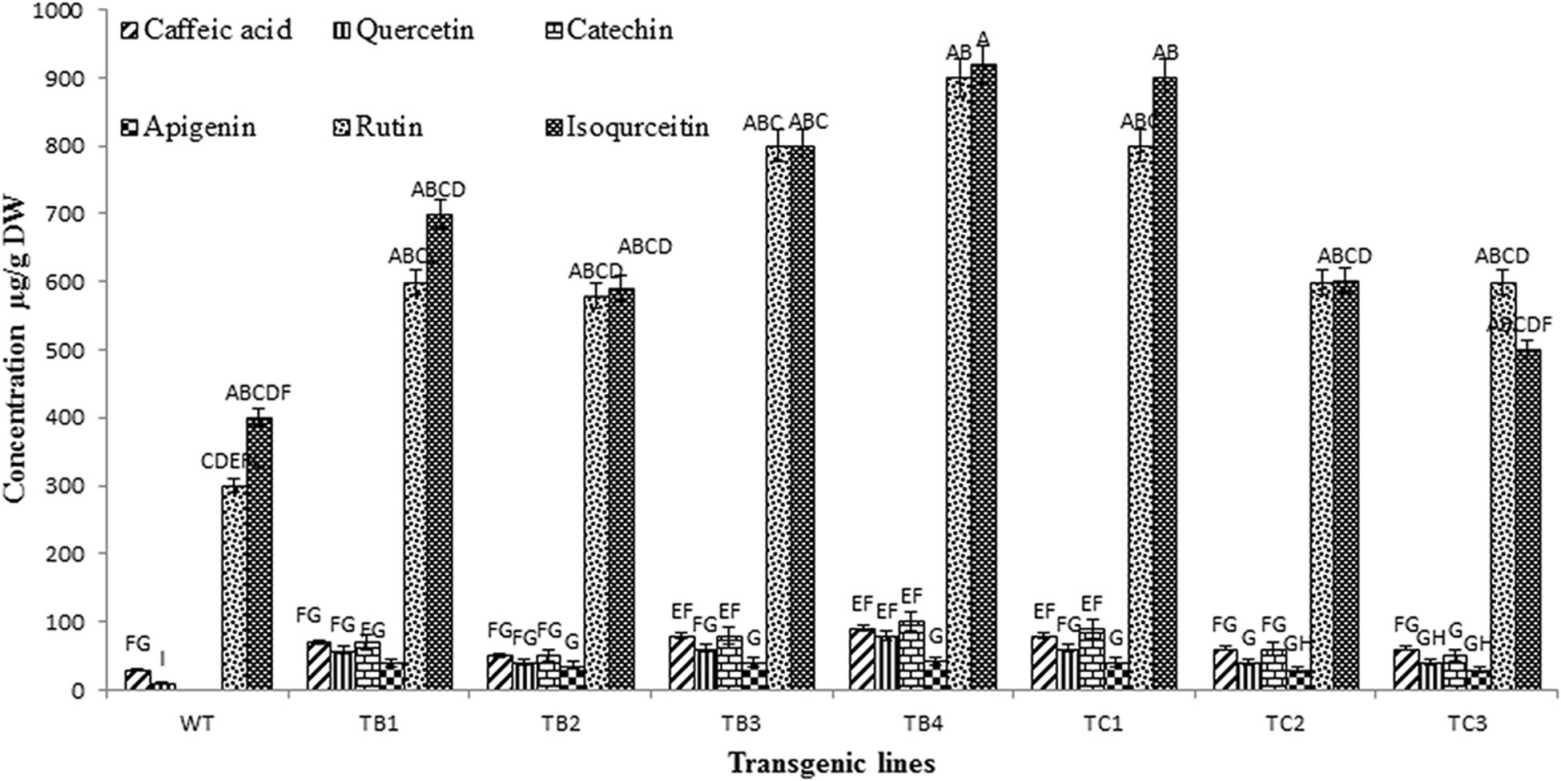
Comparative and statistical analysis of flavonoids. Each value is the mean of three replicates. Any two means having a common alphabet are not significantly different at *p* = 0.05 using LSD. *Vertical bar* represents the standard error of the 3 means. WT = wild-type plant, TB1-TB4 = *rol B* transgenics, TC1-TC3 = *rol C* gene transgenics

Qualitative and quantitative analysis of flavonoids in shoots of wild-type and transformed *A. carvifolia* plants (4-month-old) was carried out using an HPLC-DAD system. Eight flavonoid markers (caffeic acid, quercetin, isoquercetin, rutin, catechin, apigenin, gallic acid and kaempferol) were studied, out of which four (caffeic acid, quercetin, isoquercetin and rutin) were detected in the wild-type plant and with an enhanced concentration in the *rol* gene transformants. Catechin and apigenin were detected in the transformed but not the wild-type plants (Fig. 2).

While the concentration of caffeic acid was 30 μg/g DW in the wild-type plant, in the *rol B* transformants it reached 70 μg/g DW, showing a 2.4-fold increase, and in *rol C* transformants 60 μg/g DW, showing a 2-fold increase. These concentrations, and those of the following flavonoids, are the average values. The concentration of quercetin was 10 μg/g DW in the wild-type plant, increasing up to 6-fold to 59 μg/g DW in the *rol B* transgenics and 4-fold to 40 μg/g DW in *rol C* transgenics. The wild-type concentration of isoquercetin was 400 μg/g DW, increasing 1.9-fold to 770 μg/g DW in *rol B* transformants and 1.6-fold to 660 μg/g DW in *rol C* gene transformants. The concentration of rutin in the wild-type plant was 300 μg/g DW, increasing up to 2.4-fold to 720 μg/g DW in *rol B* transformants and 1.6-fold to 570 μg/g DW in *rol C* transformants.

Catechin and apigenin were detected in the transformed but not in the wild-type plants. In *rol B* and *rol C* transformants the concentration of catechin was 75 μg/g DW and 60 μg/g DW, respectively, and that of apigenin 42 μg/g DW and 30 μg/g DW, respectively. Thus, the production levels of the studied compounds in wild-type *A. carvifolia* showed a highly significant statistical difference (*P* = 0.000) in comparison with the *rol B* and *rol C* transgenics (Table 1).

**Table 1:** Analysis of variance for factors effecting the production of flavonoids in transgenics of *rol B* and *rol C* genes

Source of variation	Degree of freedom	Sum of squares	Mean square	*F*-Value	Prob.
Transgenic lines (A)	7	0.763	0.109	6.6646	0.0000
Flavonoids (B)	5	9.591	1.918	117.2688	0.0000
Transgenic lines X Flavonoids (AXB)	35	2.051	0.059	3.5824	0.0000
Error	96	1.570	0.016		
Total	143	13.976			

Coefficient of variation: 29.63 %

Polyphenols or flavonoids have been previously detected in different *Artemisia* species, as mentioned in the introduction, but in far less quantity. *Rol ABC* genes are known to be reliable enhancers of secondary metabolite production [23–25]. The effects of individual *rol* genes from the TL-DNA of *A. rhizogenes*, A4 strain, on ginsenoside production in *P. ginseng* cell cultures has been reported, with *rol C* cultures accumulating 1.8–3-fold more ginsenoside than the control plant, while *rol B* lines were not more productive [26]. However, another study found that anthraquinone production increased in *Rubia cardifolia* when transformed with the *rol B* gene. Several reports describe the mechanism of action of the *rol B* gene [17, 27, 28]. Kiani et al. (2015) observed increased flavonoid and phenolic content in *A. dubia* after transformation with a *rol ABC* gene construct [29].

### Expression of flavonoid biosynthetic pathway genes through real time qPCR

**Fig. 3:**
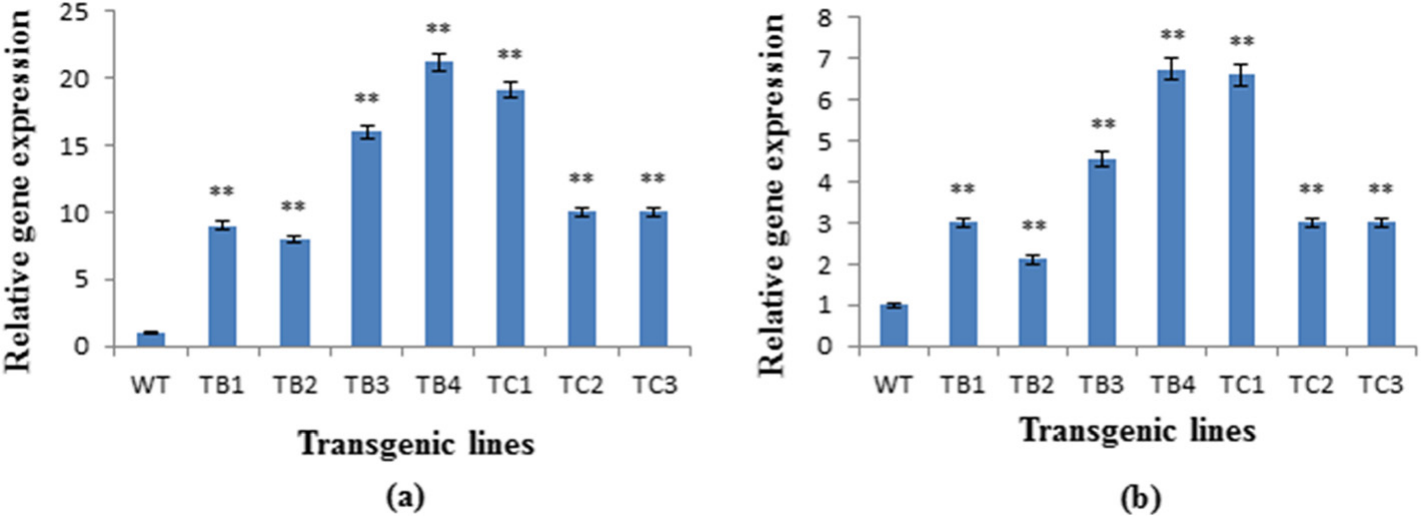
Relative expression of flavonoid biosynthetic pathway genes: Relative expression of flavonoid biosynthetic pathway genes PAL (**a**) and CHS (**b**) in wild-type *A. carvifolia* and *rol B* and *rol C* transgenics. WT = wild-type plant, TB1-TB4 = *rol B* transgenics, TC1-TC3 = *rol C* gene transgenics

Significant changes in the expression of flavonoid biosynthetic pathway genes (*PAL, CHS*) were observed in *rol* gene transgenics compared to untransformed plants (Fig. 3). The qPCR analysis clearly showed that both target genes were significantly more highly expressed (P ˂0.0001) in the transformed plants, particularly *PAL*. Increased gene expression in *rol B* transformants was 8–21-fold for *PAL* versus 3–6-fold for *CHS*. Among all the *rol* B transgenic lines, TB4 with two integrated copies of the *rol B* gene showed the highest expression of both *PAL* and *CHS*. Similarly, in *rol C* transformants expression was 10–19-fold higher for *PAL* and 3–5.8-fold higher for *CHS*, reaching a maximum in line TC1, harboring two copies of the *rol C* gene.

It has previously been described that the *PAL* enzyme catalyzes the flux of primary metabolites into the biosynthetic pathway of flavonoids through the phenylpropanoid pathway and hence performs a key role in flavonoid biosynthesis [6, 30]. *CHS*, the first enzyme of the flavonoid pathway, is an acyltransferase catalyzing the condensation of 4-coumaroyl CoA to the first flavonoid, naringenin chalcone, which is reported to be a rate-limiting step in flavonoid biosynthesis in different plants [6, 31, 32]. In walnut, *CHS* is expressed more in leaves and buds than in liber and bark and is absent from wood and medulla [33]. Various reports describe that expression of *PAL* and *CHS* is directly related to the accumulation of flavonoids in the plant tissue [34, 35]. In the current study, a positive correlation was found in the studied flavonoid accumulation and expression of the *PAL* and *CHS* genes, in agreement with previous reports [35, 36]. Other studies have also demonstrated that the overexpression of structural flavonoid biosynthetic pathway genes, including *PAL* and *CHS*, is related to an increased flavonoid accumulation pattern [36, 37].

### Evaluation of the antioxidant potential of wild-type *A. carvifolia* and *rol* gene transgenics

**Fig. 4:**
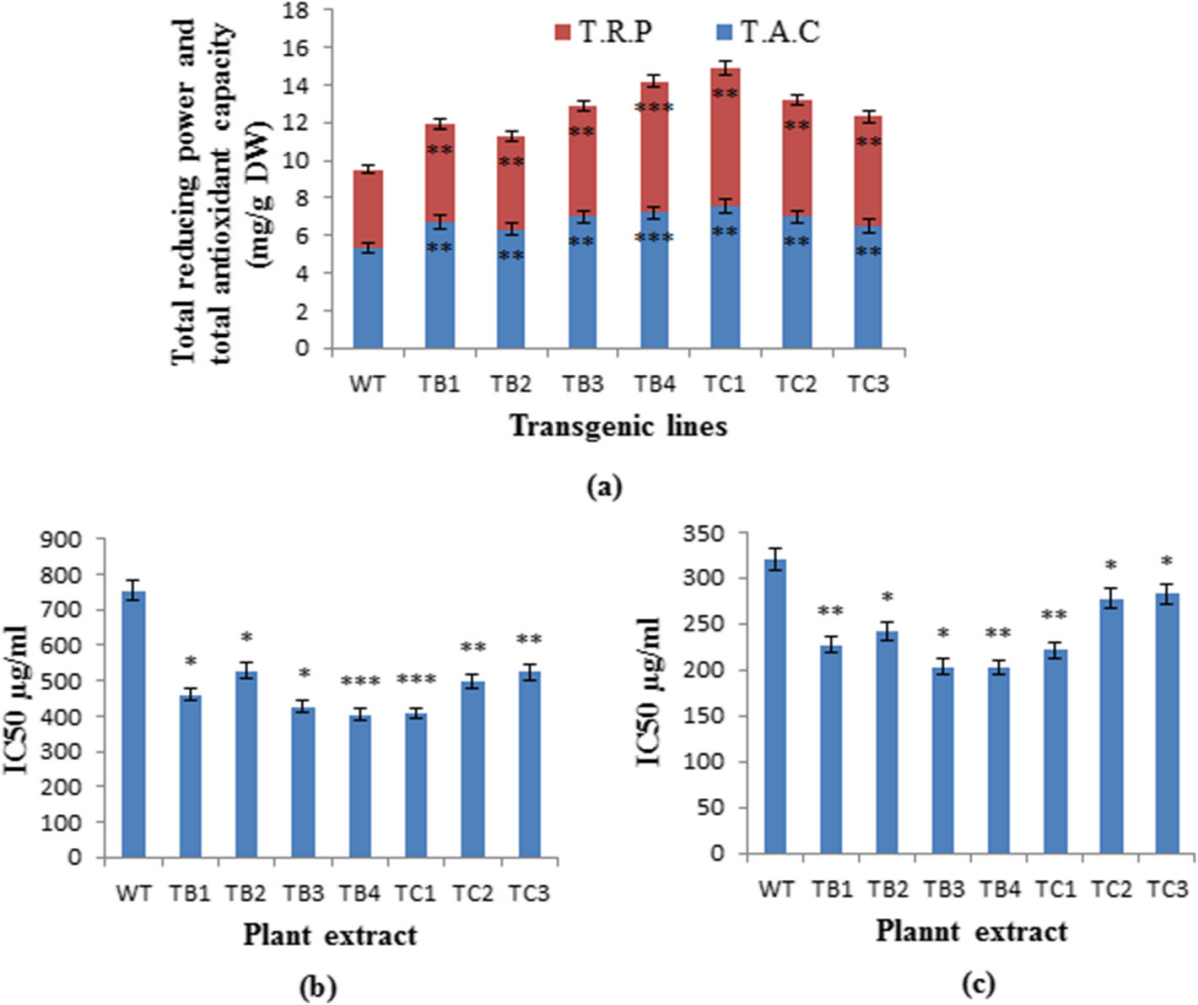
Antioxidant capacities of wild-type *A. carvifolia* and *rol B* and *rol C* transgenics. Comparison of wild-type *A. carvifolia* with transgenics of *rol B* and *rol C* genes for total reducing power and total antioxidant capacity (**a**), IC_50_ for antilipid peroxidation assay (**b**) and DPPH free radical scavenging assay (**c**). *Error bars* indicate the S.E. of triplicates. Statistical significance was measured by *t*-test (** = *P* < 0.01; * = *P* < 0.05). WT = wild-type plant, TB1-TB4 = *rol B* transgenics, TC1-TC3 = *rol C* gene transgenics

**Fig. 5:**
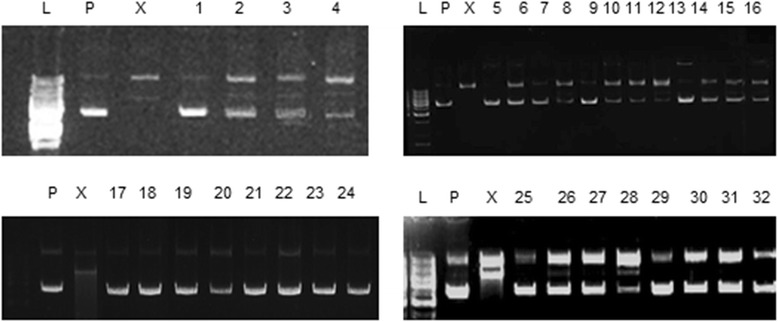
Protective effects of *A. carvifolia* wild-type and *rol B* and *rol C* transgenics against hydroxyl radical-induced DNA damage: *L* stands for 1 KB DNA ladder, “*P*” indicates plasmid DNA, *lane* X shows the damage caused to the plasmid DNA by a Fenton reaction, 1 = 1000 ppm of extract of wild-type *A. carvifolia* + plasmid DNA, 2 = 1000 ppm of extract of wild-type *A. carvifolia* + plasmid DNA + FeSO_4_ + H_2_O_2_, 3 = 100 ppm of extract of wild-type *A. carvifolia* + plasmid DNA + FeSO_4_ + H_2_O_2_, 4 = 10 ppm of extract of wild-type *A. carvifolia* + plasmid DNA + FeSO_4_ + H_2_O_2_. Similarly, *lanes* 5–8 show results of TB1, 9–12 = TB2, 13–16 = TB3, 17–20 = TB4, 21–24 = TC1, 25–28 = TC2, 29–32 = TC3

To assess the antioxidant potential of the transformed and untransformed *A. carvifolia* plants, different antioxidant assays were performed. Total antioxidant capacity, measured as the equivalent of ascorbic acid (mg/g of the DW), was 0.53 % (Fig. 4a) in the wild-type plant, compared to 0.76 % in *rol B* and 0.7 % in *rol C* gene transformants. Total reducing power was also enhanced in the transgenics, being up to 3.4 % for *rol B* and 3 % for *rol C*, compared with 2 % in the wild-type plant (Fig. 4a). Likewise, transformed plants showed lower IC50 values for anti-lipid peroxidation (Fig. 4b) and DPPH free radical scavenging activity (Fig. 4c); they were also more active in protecting the DNA against free hydroxyl radical-induced damage (Fig. 5). *Rol* genes showed highly significant effect i.e. *p* = 0.0000 on the antioxidant potential of the plant under study (Tables 2 and 3). All these findings suggest that the integration of the *rol B* and *rol C* genes enhanced the antioxidant potential of the respective transgenic lines.

**Table 2:** ANOVA for antilipid peroxidation assay

Source of variation	Degree of freedom	Sum of squares	Mean square	*F*-Value	Prob.
Concentrations (A)	2	14254.201	7127.100	8545.4007	0.0000
Genotype (B)	7	211.206	30.172	36.1766	0.0000
Concentration X Genotype (AXB)	14	246.106	17.579	21.0772	0.0000
Error	48	40.033	0.834		
Total	71	14751.546			

Coefficient of variation: 1.91 %

**Table 3:** ANOVA for DPPH free radical scavenging assay

Source of variation	Degree of freedom	Sum of squares	Mean square	*F*-Value	Prob.
Concentrations (A)	2	15107.802	7553.90	101470.30	0.0000
Genotype (B)	7	1849.574	264.22	3549.28	0.0000
Concentration X Genotype (AXB)	14	2339.980	167.14	2245.18	0.0000
Error	48	3.573	0.074		
Total	71	19300.930			

Coefficient of variation: 2.15 %

Oxidative stress is considered the root cause of the pathogenesis of many diseases, and antioxidants can be an effective treatment [38, 39]. The study of antioxidant-containing plant extracts provides insight into the mechanisms of action responsible for plant defense against oxidative damage, as well as identifying the specific antioxidant constituents [40]. The current study demonstrates that methanolic extracts of *A. carvifolia* have significant antioxidant properties.

Polyphenols are major plant antioxidants due to their redox capacity [41]. They play an important role in neutralizing or quenching free radicals and decomposing peroxides [42]. DPPH is an organic radical widely used in analyzing the antioxidant potential of pure compounds and plant extracts [43]. The reaction between the antioxidant and DPPH mainly depends on the hydrogen-donating ability, and therefore the structural confirmation, of the former [44]. The reducing power of any compound or plant extract is in fact its potential to transfer electrons, which indicates its antioxidant capacity [45]. The ferric reducing power assay is used to evaluate the antioxidant potential of dietary polyphenols [46]. The reducing capacity shown by plant extracts indicates their antioxidant activity [47].

The antioxidant properties of methanolic extracts of *Artemisia* species have been correlated with their phenolic and flavonoid content [48]. Experiments have been performed to understand the relationship between secondary metabolism activation and reactive oxygen species production (ROS) in *R. cardifolia* transformed with the *rol B* [49] and *rol C* genes [14, 50]. A significant reduction in intracellular ROS level was observed in the transformed cells of *R. cardifolia*, thus indicating that the *rol B* and *rol C* genes are potential suppressors of ROS. This decrease in ROS was also accompanied by the enhanced expression of genes encoding ROS detoxifying enzymes [49, 50].

## Conclusion

It can be concluded from the results that *rol* genes are effective in increasing flavonoid levels of *A. carvifolia* Buch, as confirmed by the HPLC-DAD analysis and enhanced antioxidant potential of *rol* gene transformants. The transgenic plants also had higher transcript levels of genes involved in flavonoid biosynthesis than the wild-type plants, which was in accordance with their higher flavonoid content. The *rol B* gene was more effective than the *rol C* gene in promoting secondary metabolism in *A. carvifolia* Buch.

## Methods

Seeds of *Artemisia carvifolia* were collected from Astore, in the Northern regions of Pakistan (35.3667° N, 74.8500° E; 8500 ft elevation) and grown on half strength MS medium. Identification of *Artemisia carvifolia* Buch was done at the Herbarium of Quaid-i-Azam University, Islamabad, Pakistan, where the specimen voucher number was submitted. After that, identification was confirmed through DNA barcoding [21]. Wild-type plants of *A. carvifolia*, as well as four *rol B* transgenic lines and three *rol C* transgenic lines produced previously [21], were analyzed for flavonoid content variation and also by real time qPCR to determine the expression of flavonoid biosynthetic genes. Additionally, the antioxidant potential of all the plants under study was determined.

### Analysis of flavonoids through an HPLC-DAD system

Extraction of flavonoids from shoots of wild-type plants and *rol* gene transgenics (4-months old) was carried out according to the reported procedure [51]. Qualitative and quantitative analysis of flavonoids was carried out using a Waters Acuity ^TM^ HPLC–DAD system (Waters, Spain) attached to a symmetry C-18 analytical column with dimensions of 5 μm, 3.9 × 150 mm (Waters, Spain). The wavelength was adjusted to 235–450 nm, and pressure applied was 200 psi. Separation was achieved using a mobile phase of acetonitrile with 0.5 % formic acid (A) and water with 0.5 % formic acid (B) running at a flow rate of 1 ml/min, with the following gradient (t (min), %B): (0, 95) (15, 65) (18, 10) (22, 95). The injection volume was 10 μl and retention time was 27 min. Peaks in extracts were identified by comparison with retention indices of reference standards. The analytes were detected at wavelengths specific for each metabolite with a particular retention time (Table 4).

**Table 4:** Retention time of studied flavonoids with wavelength

S. No	Standard	Wavelength (nm)	Retention time (min)
1	Apigenin	325	20.2
2	Caffeic acid	325	8.7
3	Catechin	279	10.7
4	Isoquercetin	355	11.1
5	Quercetin	370	15.1
6	Rutin	355	10.8

### Real time qPCR of flavonoid biosynthetic pathway genes

Expression of flavonoid biosynthetic pathway genes was studied by real time qPCR according to a previously reported method [21]. The amplification reaction was performed by gene-specific primers, i.e. *PAL* forward: 5′-ACACTCGGTTAGCTATTGCTGCAA −3′ and reverse: 5′- CCATGGCGATTTCTGCACCT −3′, *CHS* forward: 5′-AGGCTAACAGAGGAGGGTA-3′ and reverse: 5′-CCAATTTACCGGCTTTCT −3′, actin forward 5′-ATCAGCAATACCAGGGAACATAGT-3′ and reverse 5′-AGGTGCCCTGAGGTCTTGTTCC-3′.

### Measurement of antioxidant potential

The antioxidant potential of all the plants under study was determined by performing in vitro antioxidant assays. Thus, a methanolic extract of 1 g air-dried shoots was prepared after fine-grinding. Briefly, 1 g dried powdered plant material was extracted with 3 ml of methanol and subjected to sonication for half an hour. The extract was then centrifuged at 4000 g for 10 min, the supernatant was dried, and the residue was dissolved in DMSO to reach a final concentration of 100 mg/ml.

### Measurement of total antioxidant capacity

Total antioxidant capacity was determined according to the reported methodology [52] using a 96-well plate. Initially, 4 μl of the plant extract (100 mg/ml) was added to the wells and then 196 μl of the total antioxidant capacity reagent was added. After incubating the mixture for 90 min at 90 °C in a water bath, its color changed to dark blue. The mixture was then cooled and sample absorbance was taken at 630 nm on a microplate reader (Biotek, Elx 800). Ascorbic acid was used as a positive control and DMSO was used as a negative control. The total antioxidant capacity of the sample was calculated using the following formula:

$$ \mathrm{Ascorbic}\kern0.5em \mathrm{Acid}\kern0.5em \mathrm{Equivalence}=100/2.651\times \mathrm{Absorbance}\kern0.5em \mathrm{of}\kern0.5em \mathrm{sample}\kern0.5em \upmu \mathrm{g}/\mathrm{ml} $$

### Measurement of total reducing power

The total reducing power of *A. carvifolia* transformed and untransformed plant extracts was determined [53] using a 96-well plate. Twenty microlitres of plant extract (100 mg/ml) was added to the Eppendorf tubes together with 490 μl of 0.2 M phosphate buffer and 490 μL of 1 % potassium ferricyanide, which was incubated at 50 °C for 20 min. Five-hundred microlitres of trichloroacetic acid was added to the Eppendorf tubes and the mixture was centrifuged at 3000 rpm for 10 min. Five-hundred microlitres of the supernatant was isolated in a new Eppendorf tube and 100 μl of ferric chloride was added. The color of the ferric chloride changed to blue on reduction. Two-hundred microlitres of this sample was poured into the wells. Absorbance of the samples was taken at 630 nm on a microplate reader. Ascorbic acid and DMSO were used as positive and negative controls, respectively. The reducing power of the sample was calculated using the following formula:

$$ \mathrm{Ascorbic}\kern0.5em \mathrm{Acid}\kern0.5em \mathrm{Equivalence}=100/2.7025\times \mathrm{Absorbance}\kern0.5em \mathrm{of}\kern0.5em \mathrm{sample}\kern0.5em \upmu \mathrm{g}/\mathrm{ml} $$

### Anti-lipid peroxidation assay

The method for the anti-lipid peroxidation assay was adapted from Kanagalakshmi et al. [54]. Vitamin E and plain DMSO were used as positive and negative controls, respectively. Plant extracts were tested at the concentration of 1000, 100 and 10 ppm. Twenty microlitres of plant extract at each concentration was added to the liposomes in which lipid peroxidation had been induced. Incubation was carried out at 37 °C for 1 h. One milliliter of stopping solution was added to stop the reaction, which was boiled for 15 min and then cooled. Two-Hundred microlitres of the resulting solution was placed in the wells of a 96-well plate. Absorbance was measured at 532 nm. The % inhibition and IC_50_ value was calculated with TableCurve software.

### DPPH free radical scavenging assay

Free radical scavenging activity of all the plant extracts was measured according to the reported method [55] with minor modification as given below. The assay was run on four different concentrations of plant extract (1000, 500, 250 and 125 ppm) in a 96-well microplate. Briefly, 20 μl of each plant extract, or DMSO in the negative control and ascorbic acid in the positive control, was mixed with 180 μl of 0.1 mM freshly prepared DPPH solution. All the extracts at each concentration were run in triplicate. Incubation at 37 °C for half an hour was carried out for all the reaction mixtures. After that, absorbance was taken at 517 nm.

### Oxidative DNA damage analysis

A previously reported method [56] was adopted to find the antioxidant and prooxidant activity of the extracts of the plants under study. Fifty millimetre phosphate buffer was used to dissolve the pBR322 plasmid DNA to reach a concentration of 0.5 μg/3 μl. Plant extracts were analyzed at three different concentrations, i.e. 1000, 100, and 10 ppm. Plasmid pBR322 with the damaging agent, i.e. FeSO4 and H_2_O_2_, served as a positive control for DNA damage, whereas pBR322 DNA alone in phosphate buffer was used as the negative control. Incubation was done at 37 °C for 60 min. After that, samples were run on 1 % agarose gel and photographed under UV.

### Statistical analysis

All the experiments, including flavonoid extraction, HPLC analysis, real time qPCR and antioxidant assays, were performed in triplicate with the S.E. calculated. The data obtained by HPLC analysis was analyzed statistically by applying ANOVA and Duncan’s multiple range tests. Antioxidant assays were also analyzed statistically by ANOVA. The statistical significance of the results of the gene expression analysis and total antioxidant capacity and reducing power was determined by a *t*-test (*p* < 0.01 = **, *p* < 0.05 = *).

## Abbreviations

CHS: chalcone synthase
DMSO: dimethyl sulfoxide
DPPH: 2,2-diphenyl-1-picrylhydrazyl
DW: dry weight
MS: murashige and skoog
PAL: phenylalanine ammonia-lyase
ROS: reactive oxygen species
TBE: tris buffer EDTA
